# Supplementary material for: Preoperative serum lipids as novel predictors for concomitant thyroid carcinoma in Graves’ disease
Source: PeerJ. 2025 Aug 28;13:e19915. doi: 10.7717/peerj.19915 (PMC12399084; doi:10.7717/peerj.19915)
Supplement: Supplemental Information 2 [file peerj-13-19915-s002.docx]

Figure S1


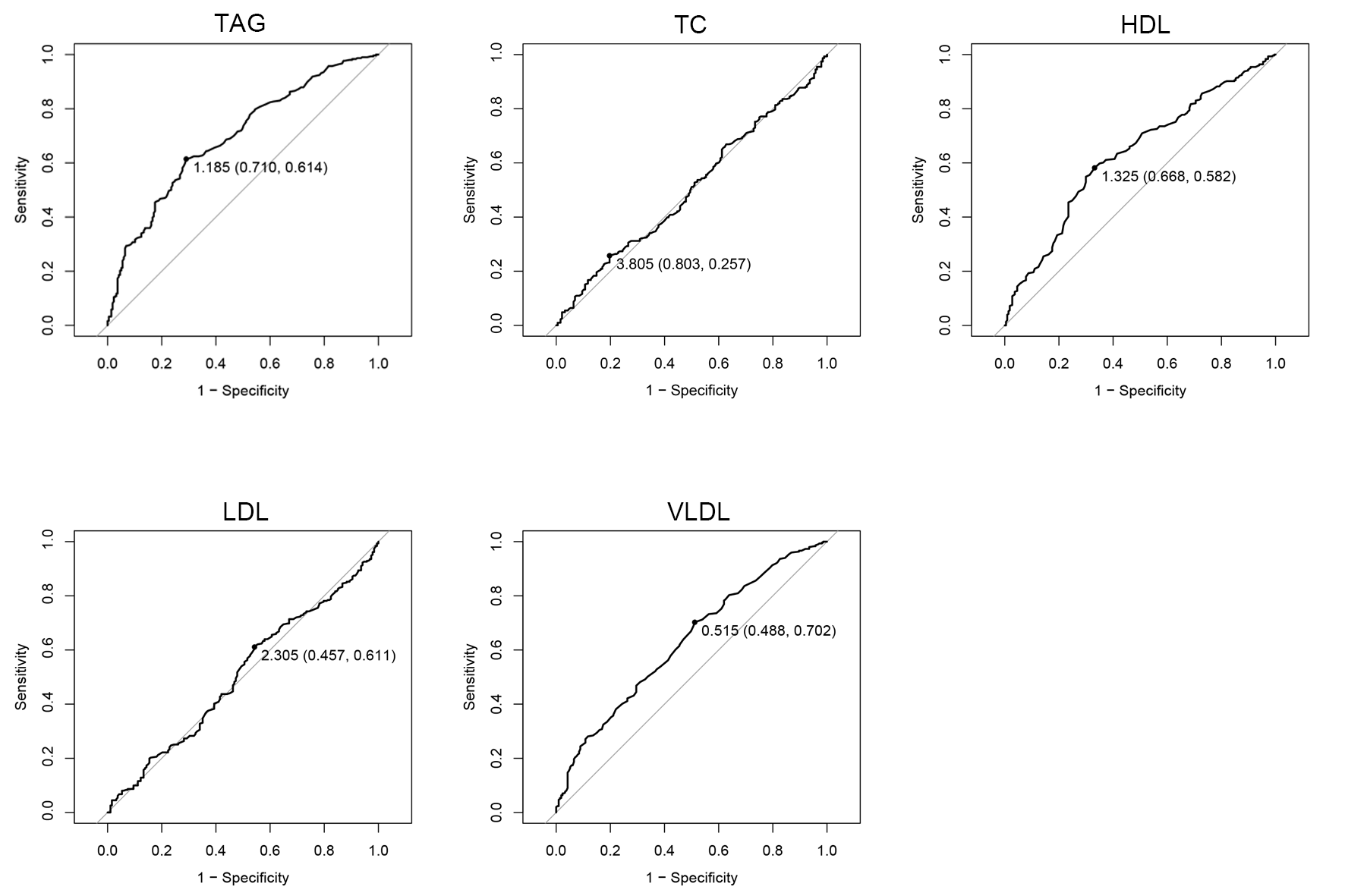


Figure S1. The cut-off value of serum TAG, TC, HDL, LDL, VLDL calculated by receiver

operating curves.

Figure S2


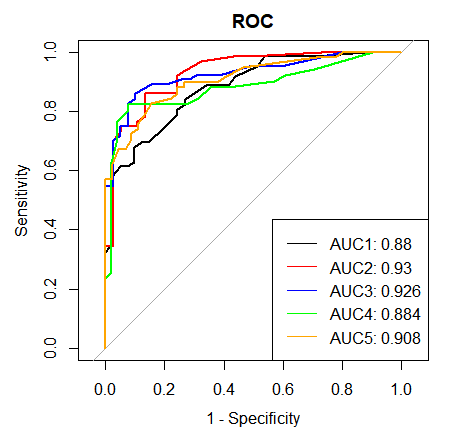


Figure S2. The ROC curve of the efficacy of nomogram model in the 5 fold-validation.

**Table S1. Baseline clinicolpathological characteristics of training and validation cohorts**

| Variables | Total (n=512) | Training cohort (n=359) | Validation cohort (n=153) | P value |
| --- | --- | --- | --- | --- |
| Age, y | 45.28 ± 13.64 | 45.17 ± 13.99 | 45.56 ± 12.83 | 0.768 |
| Gender |  |  |  |  |
| Male | 84 (16.22) | 59 (16.43) | 25 (16.34) | 0.979 |
| Female | 428 (83.59) | 300 (83.57) | 128 (83.66) |  |
| Duration of GD | 55.35 ± 79.19 | 53.79 ± 76.92 | 58.91 ± 84.33 | 0.535 |
| Smoking |  |  |  |  |
| Yes | 13 (2.54) | 10 (2.79) | 0.813 | 0.813 |
| No | 499 (97.46) | 349 (97.21) | 150 (98.04) |  |
| Drinking |  |  |  |  |
| Yes | 6 (1.17) | 6 (1.67) | 0 (0.00) | 0.246 |
| No | 506 (98.83) | 353 (98.33) | 153 (100.00) |  |
| Hypertension |  |  |  |  |
| Yes | 75 (14.65) | 55 (15.32) | 20 (13.07) | 0.510 |
| No | 437 (85.35) | 304 (84.68) | 133 (86.93) |  |
| Diabetes |  |  |  |  |
| Yes | 17 (3.32) | 10 (2.79) | 7 (4.58) | 0.592 |
| No | 495 (96.68) | 349 (97.21) | 146 (95.42) |  |
| BMI | 23.01 ± 3.39 | 22.92 ± 3.25 | 23.21 ± 3.69 | 0.384 |
| Extent of surgery |  |  |  |  |
| Lobectomy | 48 (9.38) | 31 (8.64) | 17 (11.11) | 0.379 |
| TT/near TT | 464 (90.62) | 328 (91.36) | 136 (88.89) |  |
| Cause of surgery |  |  |  |  |
| Refractory,complication to medication | 91 (17.77) | 54 (25.35) | 37 (12.37) | **<.001** |
| Huge goiter | 154 (30.08) | 136 (63.85) | 18 (6.02) |  |
| Ophthalmopathy | 26 (5.08) | 13 (6.10) | 13 (4.35) |  |
| Preoperativedx of cancer or thyroid nodule | 241 (47.07) | 10 (4.69) | 231 (77.26) |  |
| Extent of surgery |  |  |  |  |
| Lobectomy | 48 (9.38) | 22 (10.33) | 26 (8.70) | 0.532 |
| TT/near TT | 464 (90.62) | 191 (89.67) | 273 (91.30) |  |
| TgAb | 120.93 ± 266.96 | 125.13 ± 278.75 | 111.54 ± 239.07 | 0.611 |
| TPOAb | 276.20 ± 568.75 | 294.25 ± 635.51 | 232.42 ± 356.73 | 0.269 |
| Tg | 364.85 ± 1079.70 | 346.25 ± 975.57 | 403.79 ± 1273.14 | 0.592 |
| Serum lipids |  |  |  |  |
| TAG | 1.37 ± 0.93 | 1.41 ± 1.03 | 1.29 ± 0.66 | 0.206 |
| TC | 4.57 ± 1.06 | 4.50 ± 1.07 | 4.74 ± 1.03 | 0.062 |
| HDL | 1.37 ± 0.36 | 1.36 ± 0.36 | 1.40 ± 0.36 | 0.293 |
| LDL | 2.53 ± 0.76 | 2.49 ± 0.74 | 2.62 ± 0.79 | 0.070 |
| VLDL | 0.67 ± 0.49 | 0.65 ± 0.54 | 0.71 ± 0.35 | 0.212 |
| Ultrasound Characteristics |  |  |  |  |
| with nodules |  |  |  |  |
| Yes | 470 (91.80) | 326 (90.81) | 144 (94.12) | 0.212 |
| No | 42 (8.20) | 33 (9.19) | 9 (5.88) |  |
| Aspect ratio imbalance |  |  |  |  |
| Yes | 140 (27.34) | 91 (25.35) | 49 (32.03) | 0.121 |
| No | 372 (72.66) | 268 (74.65) | 104 (67.97) |  |
| Hypoechogenicity |  |  |  |  |
| Yes | 247 (48.24) | 169 (47.08) | 78 (50.98) | 0.418 |
| No | 265 (51.76) | 190 (52.92) | 75 (49.02) |  |
| Irregular borders |  |  |  |  |
| Yes | 221 (43.16) | 150 (41.78) | 71 (46.41) | 0.334 |
| No | 291 (56.84) | 209 (58.22) | 82 (53.59) |  |
| Microcalcifications |  |  |  |  |
| Yes | 41 (8.01) | 24 (6.69) | 17 (11.11) | 0.091 |
| No | 471 (91.99) | 335 (93.31) | 136 (88.89) |  |
| Irregular shape |  |  |  |  |
| Yes | 95 (18.55) | 70 (19.50) | 25 (16.34) | 0.400 |
| No | 417 (81.45) | 289 (80.50) | 128 (83.66) |  |

**Table S2 Clinical characteristics of the high HDL and low HDL groups**

| Variables | HDL | | |
| --- | --- | --- | --- |
|  | Low | High | *P* |
| Age(>55) |  |  | 0.173 |
| <55 | 117 (78.00) | 106 (71.14) |  |
| ≥55 | 33 (22.00) | 43 (28.86) |  |
| BRAF^V600E^ |  |  | 0.563 |
| No | 5 (9.62) | 7 (13.21) |  |
| Yes | 47 (90.38) | 46 (86.79) |  |
| Tumor size |  |  | 0.923 |
| <10 mm | 110 (73.33) | 110 (73.83) |  |
| ≥10 mm | 40 (26.67) | 39 (26.17) |  |
| Multifocality |  |  | 0.754 |
| No | 92 (61.33) | 94 (63.09) |  |
| Yes | 58 (38.67) | 55 (36.91) |  |
| Bilaterality |  |  | 0.051 |
| No | 104 (69.33) | 118 (79.19) |  |
| Yes | 46 (30.67) | 31 (20.81) |  |
| ETE |  |  | 0.655 |
| No | 87 (58.00) | 85 (57.05) |  |
| Minimal | 60 (40.00) | 58 (38.93) |  |
| Gross | 3 (2.00) | 6 (4.03) |  |
| LNM |  |  | 0.296 |
| No | 87 (62.14) | 94 (68.12) |  |
| Yes | 53 (37.86) | 44 (31.88) |  |
| AJCC 8th stage |  |  | 0.569 |
| Ⅰ | 141 (94.00) | 137 (91.95) |  |
| Ⅱ | 9 (6.00) | 11 (7.38) |  |
| Ⅲ | 0 (0.00) | 1 (0.67) |  |

**Table S3 Clinical characteristics of the high BMI and low BMI groups**

| Variables | BMI | | |
| --- | --- | --- | --- |
|  | Low | High | *P* |
| Age (>55) |  |  | 0.869 |
| <55 | 111 (75.00) | 112 (74.17) |  |
| ≥55 | 37 (25.00) | 39 (25.83) |  |
| BRAF^V600E^ |  |  | 0.105 |
| No | 8 (17.02) | 4 (6.90) |  |
| Yes | 39 (82.98) | 54 (93.10) |  |
| Tumor size |  |  | 0.619 |
| <10 mm | 107 (72.30) | 113 (74.83) |  |
| ≥10 mm | 41 (27.70) | 38 (25.17) |  |
| Multifocality |  |  | 0.464 |
| No | 89 (60.14) | 97 (64.24) |  |
| Yes | 59 (39.86) | 54 (35.76) |  |
| Bilaterality |  |  | 0.196 |
| No | 105 (70.95) | 117 (77.48) |  |
| Yes | 43 (29.05) | 34 (22.52) |  |
| ETE |  |  | 0.380 |
| No | 88 (59.46) | 84 (55.63) |  |
| Minimal | 54 (36.49) | 64 (42.38) |  |
| Gross | 6 (4.05) | 3 (1.99) |  |
| LNM |  |  | 0.072 |
| No | 97 (70.29) | 84 (60.00) |  |
| Yes | 41 (29.71) | 56 (40.00) |  |
| AJCC 8th stage |  |  | 0.055 |
| Ⅰ | 142 (95.95) | 136 (90.07) |  |
| Ⅱ | 5 (3.38) | 15 (9.93) |  |
| Ⅲ | 1 (0.68) | 0 (0.00) |  |

**Table S4. Serum lipids levels in GD patients with different levels of TSH**

|  |  |  |  |  |  |  |
| --- | --- | --- | --- | --- | --- | --- |
| Variables | Total  (n = 512) | TSH Low  (n = 294) | TSH Normal  (n = 167) | TSH High  (n = 51) | *P* |  |
|  |  |  |  |  |  |  |
| TAG |  |  |  |  | **0.033** |  |
| Low | 266 (51.95) | 139 (47.28) | 100 (59.88) | 27 (52.94) |  |  |
| High | 246 (48.05) | 155 (52.72) | 67 (40.12) | 24 (47.06) |  |  |
| TC |  |  |  |  | 0.533 |  |
| Low | 118 (23.05) | 72 (24.49) | 37 (22.16) | 9 (17.65) |  |  |
| High | 394 (76.95) | 222 (75.51) | 130 (77.84) | 42 (82.35) |  |  |
| HDL |  |  |  |  | 0.532 |  |
| Low | 247 (48.24) | 146 (49.66) | 80 (47.90) | 21 (41.18) |  |  |
| High | 265 (51.76) | 148 (50.34) | 87 (52.10) | 30 (58.82) |  |  |
| LDL |  |  |  |  | 0.057 |  |
| Low | 211 (41.21) | 133 (45.24) | 63 (37.72) | 15 (29.41) |  |  |
| High | 301 (58.79) | 161 (54.76) | 104 (62.28) | 36 (70.59) |  |  |
| VLDL |  |  |  |  | 0.274 |  |
| Low | 184 (35.94) | 100 (34.01) | 68 (40.72) | 16 (31.37) |  |  |
| High | 328 (64.06) | 194 (65.99) | 99 (59.28) | 35 (68.63) |  |  |

**Table S5. Serum lipids levels in GD patients with different levels of Trab**

| Variables | Total  (n = 512) | Trab Low  (n = 69) | Trab High  (n = 443) | *P* |
| --- | --- | --- | --- | --- |
|  |  |  |  |  |
| TAG |  |  |  | 0.282 |
| Low | 266 (51.95) | 40 (57.97) | 226 (51.02) |  |
| High | 246 (48.05) | 29 (42.03) | 217 (48.98) |  |
| TC |  |  |  | 0.976 |
| Low | 118 (23.05) | 16 (23.19) | 102 (23.02) |  |
| High | 394 (76.95) | 53 (76.81) | 341 (76.98) |  |
| HDL |  |  |  | 0.941 |
| Low | 247 (48.24) | 33 (47.83) | 214 (48.31) |  |
| High | 265 (51.76) | 36 (52.17) | 229 (51.69) |  |
| LDL |  |  |  | 0.349 |
| Low | 211 (41.21) | 32 (46.38) | 179 (40.41) |  |
| High | 301 (58.79) | 37 (53.62) | 264 (59.59) |  |
| VLDL |  |  |  | 0.16 |
| Low | 184 (35.94) | 30 (43.48) | 154 (34.76) |  |
| High | 328 (64.06) | 39 (56.52) | 289 (65.24) |  |

**Table S6. TSH/Trab levels in GD patients with or without thyroid cancer**

| Variables | Total (n = 512) | GD without cancer (n=213) | GD with cancer (n=299) | *P* |
| --- | --- | --- | --- | --- |
|  |  |  |  |  |
| TSH |  |  |  | 0.321 |
| Low | 294 (57.42) | 114 (53.52) | 180 (60.20) |  |
| Normal | 167 (32.62) | 76 (35.68) | 91 (30.43) |  |
| High | 51 (9.96) | 23 (10.80) | 28 (9.36) |  |
| Trab |  |  |  | 0.098 |
| Low | 69 (13.48) | 35 (16.43) | 34 (11.37) |  |
| High | 443 (86.52) | 178 (83.57) | 265 (88.63) |  |
